# A three-year longitudinal evaluation of the forearm bone density of users of etonogestrel- and levonorgestrel-releasing contraceptive implants

**DOI:** 10.1186/1742-4755-4-11

**Published:** 2007-11-12

**Authors:** Cecilia Monteiro-Dantas, Ximena Espejo-Arce, Jeffrey F Lui-Filho, Arlete M Fernandes, Ilza Monteiro, Luis Bahamondes

**Affiliations:** 1 Human Reproduction Unit, Department of Obstetrics and Gynecology, School of Medicine, Universidade Estadual de Campinas (UNICAMP), 13084-971, Campinas, SP, Brazil

## Abstract

**Background:**

The aim of this study was to evaluate bone mineral density (BMD) at baseline and at 18 and 36 months of use of etonogestrel (ENG)-and levonorgestrel (LNG)-releasing contraceptive implants. This is a continuation of a previous study in which BMD was evaluated at baseline and at 18 months of use.

**Methods:**

A total of 111 women, 19–43 years of age, wererandomly allocated to use one of the two implants. At 36 months of follow-up, only 36 and 39 women were still using the ENG- and LNG-releasing implants, respectively. BMD was evaluated at the distal and at the ultra-distal radius of the non-dominant forearm using dual-energy X-ray absorptiometry.

**Results:**

There was no difference in the BMD of users of either implant at 18 and at 36 months. BMD was significantly lower at 18 and at 36 months at the distal radius in both groups of users compared to pre-insertion values; however, no difference was found at the ultra-distal radius.

**Conclusion:**

Women 19–43 years of age using either one of these two contraceptive implants for 36 months had lower BMD values at the distal radius compared to pre-insertion values; however, no difference was found at the ultra-distal radius.

## Background

Although hormonal contraceptive methods are in use worldwide, their effect on bone mineraldensity (BMD) is a controversial issue, particularly with reference to the use of progestogen-only (p-only) methods [1]. Users of hormonal contraceptives include women of different ethnic groups and ages, ranging from adolescence through the final reproductive years. The impact of these contraceptive methods on BMD may be measured during use, after discontinuation or in the post menopause, and may also be evaluated according to bone loss or risk of osteoporotic fracture [1].

It has been well-established that hypo-estrogenism is one of the most important factors related to bone formation and resorption during a woman's life time [2,3] and it is also known that the use of some of the p-only contraceptive methods, mainly the injectable depot-medroxyprogesterone acetate (DMPA), affects serum levels of estradiol [4,5] For this reason and based on several publications showing that the use of DMPA may affect BMD [4,6-8], the US Food and Drug Administration [9] and the United Kingdom Committee on the Safety of Medicines [10] required inclusion in the package insertof DMPA of a warningthat the drug may impair BMD.

Although a recommendation issued by the World Health Organization (WHO) also established some restrictions to the use of DMPA, principally in adolescents and in women in the menopausal transition [11], it has also established that the data on levonorgestrel (LNG)-releasing implants suggest no adverse effect on BMD and recommended no restriction on the use of other p-onlycontraceptive methods by women who are eligibleto use them [11].

Since data on BMD in users of etonogestrel (ENG)- and LNG-releasing contraceptive implants is scarce [8,12-15], this study was conducted with the aim of evaluating BMD prior to insertion and following 36 months of use in women who opted to use ENG- and LNG-releasing subdermal contraceptive implants.

## Methods

This is a continuation of a previous study in which data was presented on BMD levels prior to insertion and at 18 months of use of these two types of contraceptive implant [15]. Initially, a total of 111 women were evaluated at the Human Reproduction Unit, Department of Obstetrics and Gynecology, School of Medicine, Universidade Estadual de Campinas (UNICAMP), Campinas, Brazil. The Ethical Committee of the institutionapproved the study, and all participants signed an informed consent form prior to admission.

The methods of this study have been published previously [15], together with the results of BMD at baseline and at 18 months of use. Briefly, the women comprised a subset of participantsfrom a larger study conducted by the UNDP/UNFPA/World Bank/WHO Special Programme in Research Training in Human Reproduction, World Health Organization. Women requesting an implant as a contraceptive method were randomly allocated to one ofthe two kinds of implant using a computer-generated randomization system and sealed envelopes. Fifty-six women received a single-rod, ENG-releasing implant (Implanon^®^, NV Organon, Oss, The Netherlands) and 55 women received a two^-^silicone-rod, LNG-releasing implant (Jadelle^®^, Bayer-Schering Pharma Oy, Turku, Finland). All the insertions were performedwithin the first 5 days of the menstrual cycle and there wasno wash-out time between the last contraceptive method usedand implant insertion.

Women were excluded following these criteria: pregnantor lactating within the 12 months preceding enrolment, presented chronic diseases such as diabetesmellitus, chronic renal failure, hyper/hypothyroidism, hyper/hypoparathyroidism, hepatitis, cancer or pituitary diseases and used calcium supplementation and/or vitamin D, anticonvulsants, corticosteroids, thiazide diuretics or drugs forthe treatment of thyroid disease. Nine women (four in the Implanon^® ^groupand five in the Jadelle^® ^group) were in amenorrhea at the time of insertion due to the past use of DMPA.

Based on a previous study [14], sample size was estimated at 48 women in each group assuming a difference in BMD of0.014 g/cm^2 ^between pre-insertion and 18 months of implant use, an α of 0.20, β of 0.05, and a difference of 0.008 [16].

### Definition of variables

The dependent variable, BMD, was defined as the relationship between bone mineral content (g/cm^2^) and the area of the bone measured. The independent variable was the kind of implant used by the woman. The control variables included race, number of pregnancies and deliveries, time of exclusive and partial breast-feeding, body mass index (BMI, kg/m^2^), duration of exercise practice, smoking habits [17], and coffee and alcohol consumption patterns [18].

### Bone mineral density measurement

BMD was measured in three opportunities in the non-dominant forearm at distal and ultra-distal radius with the same equipment of dual-energy X-ray absorptiometry (DXA) (DTX-200; Osteometer Meditech A/SCo., Rodovre, Denmark). Two measurements of BMD were taken in each woman: at the distal radius (where cortical bone predominates), at the point where the radius is 8 mm from the ulna; and at the ultra-distal radius near the articulation with the carpal bones (where trabecular bone predominates [65%]). The equipment identified the 8 mm distance between ulna and radius. This ensured that the same area was assessed every time. The equipment was calibrated by twice-daily phantom measurements with a device provided by the manufacturer. The calibration, positioning, assessment and calculation were performed automatically in order to minimize the operator errors. The in vivo accuracy is >97% and precision >99% and the coefficient of variation (CV) was 0.8%.

### Statistical analysis

Comparison of demographic, anthropometric and obstetric variables and BMD between the two groups was performed using Pearson and Yates' χ^2 ^tests as appropriate. To compare BMD at baseline and at 36 months, Wilcoxon's non-parametric test or Student's *t*-test was used [19]. The data are presented as mean ± standard deviation (SD).

## Results

Of the 111 women who underwent BMD measurement prior to insertion and at 18 months of implant use, only 36 and 39 women were still in use of Implanon^® ^and Jadelle^®^, respectively, at 36 months of evaluation. The decrease in the number of cases was due to premature removals. The mean age of women at implant insertion was 27.9 ± 0.7 and 27.1 ± 0.6 (mean ± SEM) years and body mass index (BMI, Kg/m^2^) was 24.0 ± 0.5 and 24.8 ± 0.5 for Implanon and Jadelle users, respectively. At 18 months of use, BMI increased significantly to 24.7 ± 0.5 in Implanon users (*P *< 0.021) and 25.6 ± 0.5in Jadelle users (*P *< 0.001) [15] and at 36 months of use was 25.4 ± 0.6 and 24.4 ± 0.6, respectively for both groups of users.

There were no significant differences at pre-insertion in number of pregnancies and deliveries in the two groups. In both groups, almost 40% of the women were housewives and ~15% were professional workers; 77% of Implanon users and 82% of Jadelle users were white. At pre-insertion, combined oral or injectable contraceptives were used by 45% and 35% in the Implanon and Jadelle groups, respectively. DMPA was used by four and five women in both groups. The remaining women were users of non hormonal contraceptive methods before implant insertion. Fourteen women in the Implanon group and 10 in the Jadelle group were in amenorrhea at 18 months of evaluation. Only two women in the Implanon^® ^group were in amenorrhea at 36 months of evaluation.

The BMD at the distal radius was significantly lower at 18 and at 36 months of use compared with baseline measurements in both Implanon^® ^users (-5.90%) (*P *< 0.001) and Jadelle^® ^users (-4.44%) (*P *< 0.002). However, at the ultra-distal radius, there were no significant differences in BMD at 18 and 36 months compared with baseline values in either group of implant users (Table 1). Moreover, there were no significant differences in BMD between users of Implanon^® ^and Jadelle^® ^at baseline or at 18 and 36 months of use.

**Table 1:** Bone mineral density according to the type of implant used, section of the forearm and duration of use.

	BMD (g/cm^2^)			
	Distal radius	*P*-value	Ultra-distal radius	*P*-value
	
*Implanon (n = 36)*				
Baseline	0.475 (0.032)		0.406 (0.057)	
18 months	0.454 (0.040)	<0.0001	0.390 (0.034)	0.1041
36 months	0.447 (0.038)	<0.0001	0.396 (0.048)	0.2491
*Jadelle (n = 39)*				
Baseline	0.474 (0.002)		0.395 (0.015)	
18 months	0.459 (0.040)	<0.0001	0.398 (0.028)	0.7087
36 months	0.453 (0.058)	0.0022	0.401 (0.072)	0.4705

All values are mean ± SD

## Discussion

Our results show that there were no significant differences in BMD at ultra-distal radius, in which trabecular bone predominates, between the users of the two types of subdermal contraceptive implants prior to implant insertion or at 18 and at 36 months of use, and these results were almost identical to those found at pre-insertion and at 18 months of use [15] albeit there was a significant reduction in BMD at the distal radius in both groups of women. These results are in agreement with those reported from a previous study [14] in which BMD was evaluated in Implanon^® ^users at pre-insertion and at 2 years of use at the spine, femoral neck, Ward's triangle, trochanter, and distal radius. They reported BMD to be similar prior to insertion and at 24 months of use, although a slight decreasein BMD was observed at the femoral neck; however, this decrease was less than one SD [20].

With respect to the group that used Implanon^®^, in theory our results were to be expected because although this implant inhibits ovulation, estrogen only decreased to early follicular phase levels at the beginning of use. Moreover, estrogen levels show a tendency to increase over the years of use and amenorrhea, due to endometrial effect [21], has been reported in ~20% of users, a higher proportion than the ~5% observed in our study group. The present data is important, since we evaluated ENG-releasing implant users at the end of the approved time of use of this implant (3 years), albeit evidence suggests that the duration of the use may be extended without replacement up to 5 years [22].

In the group of users of the LNG-releasing implant (Jadelle^®^), the results were also as expected, since luteal activity was found to increase over the years of use as serum LNG declined. Moreover, serum estradiol levels were very similar between users and non-user controls [23,24]. Additionally, our results were in agreement with those of previous longitudinal and cross-sectional studies which showed no significant differences in BMD, even in adolescents [8,12,13,25-27].

The finding that BMD was significantly lower at 18 and 36 months of use at distal radius is less important than any changes observed at ultra-distal radius in which trabecular bone predominates. This finding could be an indicator that the BMD changes through the time of use. However, the percentage of reduction on BMD at distal radius ranged only from -3.2% to -5.9% in both groups of users at 18 and 36 months of use when compared to baseline values. The strengths of our study are the fact that the users were evaluated in a longitudinal manner at pre insertion and at 18 and 36 months of use and that Implanon^® ^was compared with Jadelle^® ^The limitations of the study include the fact that the number of women was less at 36 months than at 18 months of use and that BMD was measured only atthe forearm, which is less predictive of fracture risk than the lumbar spine or the femoral neck, althoughforearm BMD also provides a good predictive value [28].

BMD evaluated at the ultra-distal radius is less predictive than BMD at the hip for hip fractures and less accurate at predicting vertebral fractures than hip or spine BMD [29]. It is stated that the relative risk (95% Confidence Interval [CI]) of fracture per SD of reduction when BMD was measured at ultra-distal radius was 1.5 (95% CI 1.3–1.8), 1.7 (95% CI 1.5–1.9), and 1.7 (95% CI 1.4–2.0) for hip, vertebral, and forearm, respectively [28]. BMD measured at the femoral neck or total hip is a strong predictor of hip fracture and predicts risk of all fractures in comparison to peripheral BMD measure [28]. Peripheral measurements could beused as an initial screening test and those women with low BMD will need to be referred for BMD of the hip or spine. In a recent systematic review on the use of steroidal contraceptives and bone fractures in women [30] it was stated that there were no trials with fracture as outcome and that decreased BMD was observed only among users of DMPA with inconsistent results for implants users.

Furthermore, since the participating women were young the possibility of BMD being affected was very low mainly if we consider that BMD is a very soft clinical outcome and fracture is the most important indicator of bone health. BMD must be considered a continuous risk factor during lifetime and the lowerthe BMD the higher the risk of fracture. The estimates percentage of lifetime risk of hip fracture for white women at the age of 50 at the following three T-scores: zero, -2.0, and -3.5 were 10%, 27%, and 49%, respectively. However, these estimative were different for BMD at forearm or spine [31].

In addition, 36 months of use may be too short a time, since the effects of hormonal contraceptives may be observed over many years of use and following discontinuation, although it has been suggested that the effect of the past use of p-only contraceptives on BMD may be eliminated following discontinuation of use [8,32].

## Conclusion

Our data do not allow conclusions to be drawn about the effect of these implants on adolescents or in women in the menopausal transition. The reduction observed at the distal radius, although within the limit of 1 SD, must be analysed with caution because it is not possible to conclude whether this loss has any clinical significance over long-term use or if it has any effecton postmenopausal fracture risk [33]. Additionally, it is important to take into account that many women use hormonal contraceptive methods for a short period of time and consequently any deleterious effect could be counterbalance by a recovery after discontinuation. In conclusion, BMD was significantly lower at 18 and at 36 months of use compared to pre insertion values in users of both contraceptive implants at the distal radius; however, no differences were found at the ultra-distal radius. These results could be indicating apparently a non severe impact on BMD. This cohort of women is currently being followed-up and BMD will be measured again at 60 months of use if the number of users remains adequate at that time.

## Abbreviations

**BMD: **Bone mineral density

**BMI: **Body mass index

**CV: **Coefficient of variation

**DEXA: **Double X-ray absorptiometry

**DMPA: **Depot medroxyprogesterone acetate

**ENG: **Etonogestrel

**LNG: **Levonorgestrel

**UNDP: **United Nations Development Programme

**UNFPA: **United Nations Fund for Population Affaires

**UNICAMP: **Universidade Estadual de Campinas

**SD: **Standard deviation

**WHO: **World Health Organization

## Competing interests

The author(s) declare that they have no competing interests.

## Authors' contributions

CM-D, XEA, JFL-Fi, AMF, IM, LB contributed equally because all made substantive intellectual contributions to conception and design, acquisition of data, analysis, and interpretation and have been involved in the writing of the manuscript and have given final approval of the version submitted for publication.

## References

[B1] Curtis KM, Martins SL (2006). Progestogen-only contraception and bone mineral density: a systematic review. Contraception.

[B2] Riggs BL, Melton LJ (1986). Involutional osteoporosis. N Engl J Med.

[B3] Recker RR, Davies KM, Hinders SM, Heaney RP, Stegman MR, Kimmel DB (1992). Bone gain in young adult women. JAMA.

[B4] Gbolade B, Ellis S, Murby B, Randall S, Kirkman R (1998). Bone density in long-term users of depot medroxyprogesterone acetate. Br J Obstet Gynaecol.

[B5] Bahamondes L, Trevisan M, Andrade L, Marchi NM, Castro S, Faundes A (2000). The effect upon the human vaginal histology of the long-term use of the injectable contraceptive Depo-Provera. Contraception.

[B6] Cundy T, Evans M, Roberts H, Wattie D, Ames R, Reid IR (1991). Bone density in women receiving depot medroxyprogesterone acetate for contraception. BMJ.

[B7] Tang OS, Tang G, Yip P, Li B, Fan S (1999). Long-term depot-medroxyprogesterone acetate and bone mineral density. Contraception.

[B8] Petitti DB, Piaggio G, Mehta S, Cravioto MC, Meirik O (2000). Steroid hormone contraception and bone mineral density: a cross-sectional study in an international population. The WHO Study of Hormonal Contraception and Bone Health. Obstet Gynecol.

[B9] United States Food and Drug Administration Talk Paper Black Box Warning Added Concerning Long-Term Use of Depo-Provera Contraceptive Injection.

[B10] Duff G Updated prescribing advice on the effect of Depo-Provera contraception on bones HSS. http://www.mhra.gov.uk/home/idcplg?IdcService=GET_FILE&dDocName=con019478&RevisionSelectionMethod=Latest.

[B11] D'Arcangues C (2006). WHO statement on hormonal contraception and bone health. Contraception.

[B12] Taneepanichskul S, Intaraprasert S, Theppisai U, Chaturachinda K (1997). Bone mineral density during long-term treatment with Norplant implants and depot medroxyprogesterone acetate. A cross-sectional study of Thai women. Contraception.

[B13] Di X, Li Y, Zhang C, Jiang J, Gu S (1999). Effects of levonorgestrel-releasing subdermal contraceptive implants on bone density and bone metabolism. Contraception.

[B14] Beerthuizen R, van Beek A, Massai R, Makarainen L, Hout J, Bennink HC (2000). Bone mineral density during long-term use of the progestagen contraceptive implant Implanon compared to a non-hormonal method of contraception. Hum Reprod.

[B15] Bahamondes L, Monteiro-Dantas C, Espejo-Arce X, Dos Santos Fernandes AM, Lui-Filho JF, Perrotti M, Petta CA (2006). A prospective study of the forearm bone density of users of etonogestrel- and levonorgestrel-releasing contraceptive implants. Hum Reprod.

[B16] Pocock SJ (1987). Clinical Trials: A Practical Approach.

[B17] Kanis JA, Johnell O, Oden A, Johansson H, De Laet C, Eisman JA, Fujiwara S, Kroger H, McCloskey EV, Mellstrom D, Melton LJ, Pols H, Reeve J, Silman A, Tenenhouse A (2005). Smoking and fracture risk: a meta-analysis. Osteoporos Int.

[B18] Kanis JA, Johansson H, Johnell O, Oden A, De Laet C, Eisman JA, Pols H, Tenenhouse A (2005). Alcohol intake as a risk factor for fracture. Osteoporos Int.

[B19] Armitage P (1991). Statistical Methods in Medical Research.

[B20] World Health Organization (1994). Assessment of Fracture Risk and its Application to Screening for Post-menopausal Osteoporosis: Report of a WHO Study Group. WHO, Geneva (WHO technical report series 843).

[B21] Croxatto HB, Urbancsek J, Massai R, Coelingh Bennink H, van Beek A (1999). A multicentre efficacy and safety study of the single contraceptive implant Implanon. Implanon Study Group. Hum Reprod.

[B22] Croxatto HB, Makarainen L (1998). The pharmacodynamics and efficacy of Implanon. An overview of the data. Contraception.

[B23] Croxatto HB, Diaz S, Pavez M, Brandeis A (1988). Estradiol plasma levels during long-term treatment with Norplant subdermal implants. Contraception.

[B24] Croxatto HB (2000). Progestin implants. Steroids.

[B25] Diaz S, Reyes MV, Zepeda A, Gonzalez GB, Lopez JM, Campino C, Croxatto HB (1999). Norplant implants and progesterone vaginal rings do not affect maternal bone turnover and density during lactation and after weaning. Hum Reprod.

[B26] Naessen T, Olsson SE, Gudmundson J (1995). Differential effects on bone density of progestogen-only methods for contraception in premenopausal women. Contraception.

[B27] Cromer BA, Blair JM, Mahan JD, Zibners L, Naumovski Z (1996). A prospective comparison of bone density in adolescent girls receiving depot medroxyprogesterone acetate (Depo Provera), levonorgestrel (Norplant), or oral contraceptives. J Pediatr.

[B28] Marshall D, Johnell O, Wedel H (1996). Meta-analysis of how well measures of bone mineral density predict occurrence of osteoporotic fractures. BMJ.

[B29] Siris ES, Miller PD, Barrett-Connor E, Faulkner KG, Wehren LE, Abbott TA, Berger ML, Santora AC, Sherwood LM (2001). Identification and fracture outcomes of undiagnosed low bone mineral density in postmenopausal women: results from the National Osteoporosis Risk Assessment. JAMA.

[B30] Lopez LM, Grimes DA, Schulz KF, Curtis KM (2006). Steroidal contraceptives: effect on bone fractures in women. Cochrane Database Syst Rev.

[B31] Cummings SR, Bates D, Black DM (2002). Clinical use of bone densitometry: scientific review. JAMA.

[B32] Orr-Walker BJ, Evans MC, Ames RW, Clearwater JM, Cundy T, Reid IR (1998). The effect of past use of the injectable contraceptive depot medroxyprogesterone acetate on bone mineral density in normal post-menopausal women. Clin Endocrinol (Oxf).

[B33] Cefalu CA (2004). Is bone mineral density predictive of fracture risk reduction?. Curr Med Res Opin.

